# Controlled fluorescence quenching by antibody-conjugated graphene oxide to measure tau protein

**DOI:** 10.1098/rsos.171808

**Published:** 2018-04-11

**Authors:** Ao Huang, Luning Zhang, Weiwei Li, Zeyu Ma, Shi Shuo, Tianming Yao

**Affiliations:** School of Chemical Science and Engineering, Tongji University, 1239 Siping Road, Shanghai 200092, People's Republic of China

**Keywords:** tau protein, Alzheimer's disease, biosensor, graphene oxide, immunoassay

## Abstract

We report an ultrasensitive immunoassay for tau protein—a key marker of Alzheimer's disease. This sensing platform relies on graphene oxide (GO) surfaces conjugated with anti-human tau antibody to provide quantitative binding sites for the tau protein. The GO quenches standard fluorescein isothiocyanate labelled tau (tau-FITC) when tau protein and tau-FITC are both present and compete for the binding sites. This change in fluorescence signal can be used to quantitate tau protein. In contrast with traditional enzyme-linked immunosorbent assay (ELISA), our method does not require enzyme-linked secondary antibodies for protein recognition nor does it require an enzyme substrate for optical signal generation. This requires fewer reagents and has less systematic error than the antigen–antibody recognition steps in ELISA. Our method has a tau protein detection limit of 0.14 pmol ml^−1^ in buffer. This approach could be developed into a promising biosensor for the detection of tau protein and may be useful in the clinical diagnosis of tau-induced neurodegeneration syndromes.

## 1. Introduction

There will soon be at least 100 million people worldwide suffering from Alzheimer's disease (AD) [1], which is a neurodegenerative disorder causing serious problems like memory loss, irritability, aggression, mood swings, etc. [2,3] Despite these huge problems, there is no definitive diagnosis for AD other than traditional neuropsychological, cognitive and neurological tests; however, the accuracy of these tests depends on the cooperation of both patient and brain surgeon with only 85% accuracy [4]. Tau protein plays a very important role in the onset of AD—abnormally aggregated tau protein oligomers and paired helical filaments (PHFs) are major elements that confer cellular toxicity [5–8]. As a result, quantitative detection of tau protein can be used as a clinical diagnostic for AD [9–11].

The current approach to measuring tau protein is enzyme-linked immunosorbent assay (ELISA), but it has several shortcomings. The accuracy of ELISA is affected by a set of problems intrinsic to the technique: the uncertainty of the recognition between secondary antibodies and antigens causing false signals [12–14]; lack of definitive chemical surface properties of the ELISA 96-well polystyrene plates for protein attachment [15]; and introduction of systematic and human errors in the procedures, such as plate washing.

Recently, many research groups have tried to improve the accuracy of ELISA by introducing monoclonal antibodies [16,17], synthesizing new ELISA polymer substitutes [15], employing more sensitive biomarkers [18] and using nanomaterials [19–23]. However, these studies were optimized within the framework of traditional ELISA and still relied on ELISA plates. Thus, these problems remain largely unresolved. New techniques for immunosorbent assays call for quantitative surface sites for antigen–antibody binding and fewer steps or reagents in the process to avoid these limitations.

Graphene oxide (GO) has recently attracted much attention because of its unique structural, mechanical and electronic properties. GO is an energy acceptor with long-range energy transfer and biocompatibility. Many research groups have presented GO-based biosensors to detect metal ions [24,25], biomolecules [26–28] and viruses [29]. In these previous works, GO acted as a fluorescence quencher based on the photo-induced electron transfer mechanism or electronic energy transfer mechanism without further modification. The biomolecules were attached on a GO surface through π–π stacking and hydrophobic interactions but had no specificity, i.e. any protein that had an aromatic ring could be adsorbed by GO. The surface modification of GO by antibodies could provide better specificity through antibody–antigen reactions, but this has rarely been reported.

We recently reported [30] a GO-based biosensor suitable for detecting proteins. We validated this approach with a generic IgG analyte. We showed that the immune reaction had specificity and that energy transfer on the antibody-conjugated GO surface could be used to quantify IgG. Here, we extend this sensing approach to tau protein sensing and demonstrate that the performance metrics are compatible with clinical needs for the diagnosis of AD. Here, the antibody-conjugated GO surface can specifically bind with both regular tau protein (analyte) and fluorescent tau-FITC (standard). Thus, it exhibits competitive binding of the two types of tau proteins. The amount of analyte tau protein controls the adsorption of tau-FITC, and hence the degree of its fluorescence quenching by GO. This change in fluorescence signal is used to measure tau in the samples via a calibration curve. In clinical practice, human cerebrospinal fluid (CSF) samples are usually tested to measure tau concentrations. According to the literature [9,10], baseline tau protein in human CSF is usually 0–100 pg ml^−1^. This increases to hundreds or even thousands of pg ml^−1^ in disease (Oka *et al*. [31] reported values as high as 6.3 ng ml^−1^) which is close to the limit of detection (LOD) achieved in this study (6.4 ng ml^−1^).

**Figure 1.**
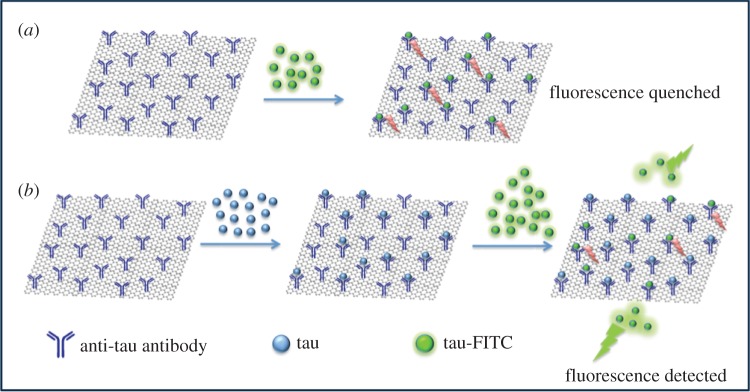
Schematic of GO-based fluorescence quenching for the detection of tau protein. (*a*) The fluorescence signal of tau-FITC will be quenched when there is no analyte tau in solution. (*b*) When analyte tau protein molecules are present and adsorb on antibody-conjugated GO, fewer tau-FITC are adsorbed and quenched. Thus, the fluorescence signal from free tau-FITC in buffer correlates with analyte tau concentration.

Figure 1*a* shows that the tau-FITC is adsorbed strongly and is quenched when there is no tau analyte in the solution. When tau is present and competes for the available binding sites, fewer tau-FITC are adsorbed on the remaining sites. They remain free in solution, and this increases the fluorescence signal (figure 1*b*). Thus, the fluorescence intensity increases with increasing tau. Next, we briefly outline the experimental methods and then present details followed by discussion and concluding remarks.

GO was first chemically modified with an anti-tau antibody via a peptide bond between the surface carboxyl groups and the amino groups of the antibody. This surface provides antibody-mediated specific binding sites, which are superior to other types of surfaces that rely on physical adsorption. The analyte tau441 proteins (i.e. the longest isoform of human tau protein [4], abbreviated as analyte tau below. This isoform is more representative than others because it contains all microtubule-associated groups related to the formation of PHFs [6]. It was added at increasing concentrations to buffer solutions containing antibody-conjugated GO. After adsorption of analyte tau proteins on some of the binding sites, FITC-labelled tau441 (abbreviated as tau-FITC) was also added. The GO surface is not only a nano-scale binding platform, but is also an energy acceptor [32–35] that quenches the fluorescence of tau-FITC. Because quenching occurs in close proximity, only the tau-FITC adsorbed on the antibody-conjugated GO became quenched [36–39]. As the number density of binding sites was limited to the modified GO, more adsorbed tau analyte resulted in stronger fluorescence signal from free tau-FITC in the solutions. Essentially, the analyte tau proteins control the fluorescence quenching and determine the fluorescence intensity.

## 2. Material and methods

### 2.1. Materials and reagents

Tau, tau-FITC, rabbit anti-human tau antibody and bovine serum albumin (BSA) were purchased from Sangon Biotech Co. Ltd (Shanghai, China). GO was purchased from XFNANO (Nanjing, China).

*N*-(3-dimethylaminopropyl)-*N*-ethylcarbodiimde hydrochloride (EDC), 2-(*N*-Morpholino) ethanesulfonic acid (MES) and *N*-hydroxysuccinimide (NHS) were purchased from Tokyo Chemical Industry (Japan). All other reagents were purchased from J&K Scientific (China) and were of analytical reagent grade and used without further purification.

### 2.2. Characterization methods

The fluorescence intensity of each sample was measured under the excitation wavelength of 490 nm with a slit width of 10 nm using a fluorescence spectrophotometer (Hitachi F-7000, Japan). The emission peak of FITC is centred at 520 nm, and thus we used the corresponding filters to obtain the needed spectral detail. For atomic force microscope (AFM) measurements, sample solutions containing bare GO or modified GO were pipetted onto freshly cleaved mica and dried in ambient air. Surface topographic features were scanned in contact mode using a commercial AFM (CSPM 4000, Benyuan, China) equipped with a silicon cantilever.

### 2.3. Preparation of rabbit anti-human tau conjugated graphene oxide

Antibody-conjugated GO was synthesized by a classic two-step EDC–NHS method [39,40]. Briefly, 1 mg GO was dispersed in 5 mmol l^−1^ MES buffer (pH = 4.0) in an ultrasonic bath. Next, a MES buffer solution containing 4 mg ml^−1^ EDC and 6 mg ml^−1^ NHS was added into the GO-dispersed MES solution to activate the GO surface. The mixture was stirred for 30 min at 10°C and then centrifuged and washed with 20 mmol l^−1^ phosphate buffer solution (PBS, pH = 7.4) to remove unreacted coupling reagents. The activated GO was again redispersed in PBS to react with 50 µg rabbit anti-human tau antibody so as to modify the GO with antibody. The samples were mixed on an electronic shaker at 10°C for 2 h. The remaining GO-active sites were blocked with 2% BSA solution in a 20 mmol l^−1^ PBS buffer for 30 min. The solution was then centrifuged at 16 000 r.c.f. for 10 min to remove any unbound biomolecules in the supernatant. About 90% of the antibodies were immobilized on the GO surface (electronic supplementary material, figure S2).

### 2.4. Process for tau protein assaying

First, a series of human tau concentrations (0–600 ng ml^−1^) was added to solutions containing 100 µg ml^−1^ antibody-conjugated GO and reacted for 1 h either at 37°C or room temperature (electronic supplementary material, figure S4). In our case, the sample was held at 37°C to simulate the human body. Afterwards, tau-FITC standard was added to each sample to reach a concentration of 100 ng ml^−1^ and allowed to react for another 1 h at the same temperature. When the reaction ended, the fluorescence intensity of each sample was measured. Response curves for the assay were obtained by plotting tau-FITC's fluorescence-intensity change as a function of the analyte tau concentration. Methods for determining the LOD and selectivity of the assay were the same as above.

## 3. Results and discussion

We first present the experimental evidence for GO morphology change upon surface modification. We then present a series of fluorescence spectra of different reaction mixtures for the characterization and optimization of assay parameters such as the quenching effect of tau-FITC on bare GO, on antibody-conjugated GO and on BSA-blocked GO surface. Finally, we show how analyte tau can be detected across a wide range of concentrations based on the fluorescence-signal change of tau-FITC. The limit of detection and also selectivity of the assay are given.

### 3.1. Morphology of antibody-conjugated graphene oxide

The antibody was conjugated to GO by a classic two-step EDC–NHS method. The carboxyl on GO (electronic supplementary material, figure S1 shows the infrared spectrum of GO and the evidence of carboxyl) was first activated by EDC and formed a mediator with NHS. Amino groups from the antibody could then couple with the mediator to form a peptide bond. To confirm the immobilization of antibodies on GO nanosheets, we used AFM to measure the surface morphologies of bare GO, EDC–NHS activated GO and antibody-conjugated GO. This provides evidence for the EDC–NHS coupling reaction and the immobilization of antibodies on GO. Lee *et al*. [40] and Hosseini *et al*. [15] showed that the relative height of GO (measured from top to bottom) would increase after activation and conjugation. These previous studies are reference points. We used AFM to characterize the antibody-modified GO and confirm the presence of antibodies on GO surface.

**Figure 2.**
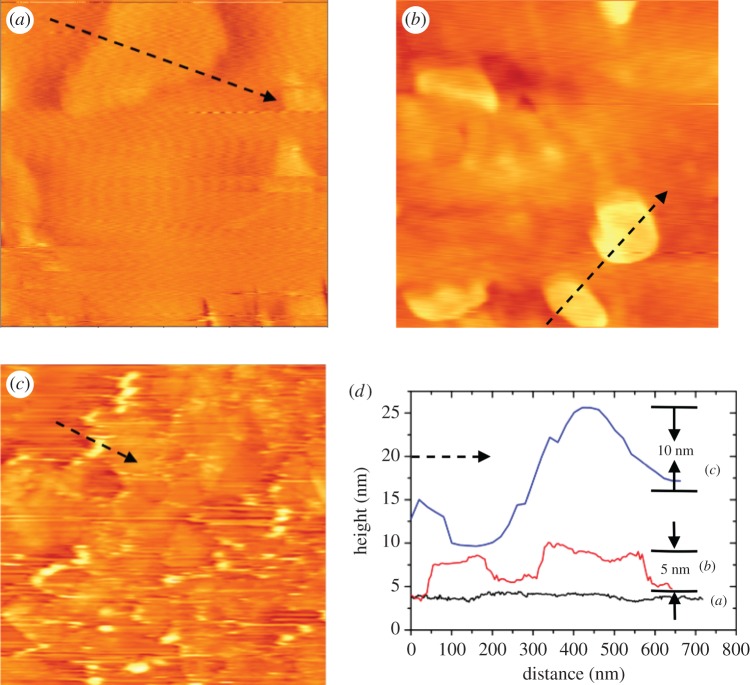
Surface morphologies of GO measured by AFM: (*a*) bare GO (approx. 0.9 nm), (*b*) EDC–NHS activated GO (approx. 5 nm), (*c*) antibody-conjugated GO (approx. 10 nm) on freshly cleaved mica, (*d*) height profiles of the line scans (arrows) in (*a*), (*b*) and (*c*). Typical surface feature relative heights (from top to bottom) are given in parentheses. Image sizes: (*a*) 1 × 1 µm; (*b*) 0.8 × 0.8 µm; (*c*) 5 × 5 µm.

In figure 2*a*, the bare GO has a flakey appearance with a height of approximately 0.9 nm on mica. This corresponds to a monolayer of GO. The corresponding line scan and height profile of the sample are shown in figure 2*d*. After being activated by EDC–NHS, GO exhibited a relative thickness of about 5 nm (from top to bottom) as shown in figure 2*b*, which is similar to values reported previously [15,40]. The line scan denoted by the arrow gave a corresponding cross-sectional height profile in figure 2*d*. This shows a fairly uniform surface of the GO. The anti-tau antibody is a large macromolecule with a molecular weight of over 150 kDa and it will make the GO much thicker.

Indeed, the height of the antibody-conjugated GO increased dramatically (figure 2*c*) with a typical thickness of about 10 nm as suggested in the height profile in figure 2*d*. Some bright spots were observed in this sample similar to Lee *et al*. [40]. The bright spots are probably due to some degree of aggregation of antibodies on the edge of the GO where higher densities of carboxyl groups might cause more antibodies to accumulate. The surface morphology results implied that the anti-tau antibody was successfully immobilized on the GO surface.

### 3.2. Determining the optimal concentration of graphene oxide

The key design of our immunoassay is quenching of tau-FITC adsorbed on antibody-conjugated GO leaving the free tau-FITC in the solution to generate fluorescence. Owing to the limited number of binding sites on antibody-conjugated GO, when more analyte tau adsorbs on the GO, fewer binding sites remain for tau-FITC. Thus, more free tau-FITC will remain in solution and will produce more fluorescence. Therefore, there is a positive and quantitative correlation between analyte tau concentration and the fluorescence intensity of free tau-FITC. The assay relies on the effectiveness of GO as an energy acceptor to provide efficient fluorescence quenching. The modification of the GO surface may change the molecular orbitals of GO and affect quenching efficiency [40]; i.e. the change in GO's quenching efficiency was determined empirically. Though we have already discussed the modification process [30], we had to study the quenching efficiency every time because we changed the recognition antibody used to sense the analyte target protein.

**Figure 3.**
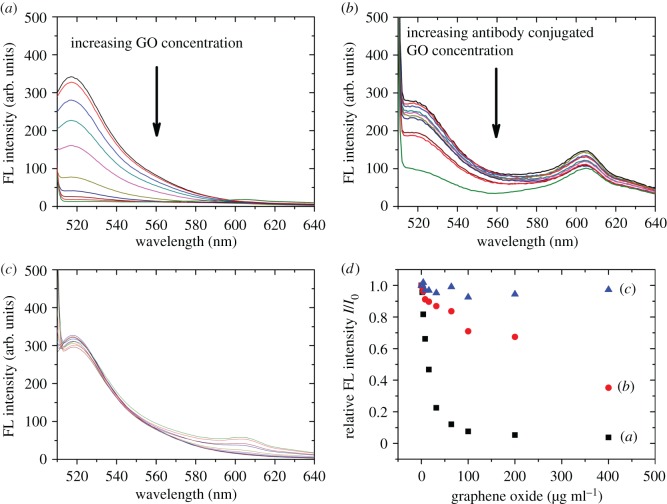
Fluorescence (FL) intensity profile of (*a*) 100 ng ml^−1^ tau-FITC reacts with an increasing concentrations of GO; (*b*) 100 ng ml^−1^ tau-FITC reacts with an increasing concentrations of antibody-conjugated GO; (*c*) 100 ng ml^−1^ tau-FITC reacts with an increasing concentration of BSA-blocked GO. The GO concentrations were 0, 2, 4, 8, 16, 32, 64, 100, 200 and 400 µg ml^−1^. The relationship between GO concentration and FL intensity of (*a*), (*b*) and (*c*) is shown in (*d*) for comparison.

Thus, we investigated the quenching efficiency of tau-FITC by bare GO, antibody-conjugated GO and BSA-blocked GO. These three types of GO were added to standard samples containing 100 ng ml^−1^ of tau-FITC. As expected, the fluorescence of tau-FITC should decrease when tau-FITC binds to the GO surfaces. Figure 3*a* shows a rapid decrease in fluorescence intensity with increasing amounts of bare GO. This then exhibits a quenching efficiency of about 90% with 100 µg ml^−1^ of GO. The signal change versus GO concentration is plotted in figure 3*d*. The transfer of energy from the excited FITC to bare GO is actually much easier than that to antibody-conjugated GO because the modification of GO with antibodies changed the electronic properties of GO turning GO into a semiconductor [40]. This implies that the quenching efficiency of antibody-conjugated GO might be lower than bare GO.

In figure 3*b*, the fluorescence intensity also decreased with the increasing amounts of antibody-conjugated GO; the quenching efficiency reached about 60% at 400 µg ml^−1^ showing that antibody-modified GO could still be a quencher. With 100 µg ml^−1^ of antibody-conjugated GO, about 30% of the tau-FITC initial fluorescence was quenched (figure 3*d*). Therefore, with analyte tau in solution, the fluorescence-intensity change in tau-FITC is between 0 and 30% with respect to initial fluorescence with no analyte. In addition, there was a fluorescence peak near 605 nm (figure 3*b*,*c*). This could not be seen in figure 3*a*. The fluorescence peak at 605 nm was due to scattering caused by antibody-conjugated GO as we suspected. The thickness of the antibody-conjugated GO was much larger than bare GO as shown by AFM. This affected the optical properties of the analyte solution and resulted in a scattering peak at 605 nm. (This peak could be eliminated after the sample was centrifuged; see electronic supplementary material, figure S3.)

One final assay characterization step was to determine if the diminishing fluorescence intensity could originate from other sources. This assay would be useless if the fluorescence of tau-FITC was quenched regardless of its free/bound state. By blocking the GO surface with 2% BSA in buffer, we found that the fluorescence intensity of tau-FITC remains nearly constant with increasing amounts of BSA-blocked GO as clearly shown in figures 3*c*,*d*. This confirms that free tau-FITC in solution would not be quenched by the presence of large concentrations (up to 400 µg ml^−1^) of inactive GO. Only the tau-FITC adsorbed on the surface of GO could be quenched.

The appropriate concentration of antibody-conjugated GO for quenching a solution containing 100 ng ml^−1^ tau-FITC was 100 µg ml^−1^ (approx. 30% quenching). Although more GO might quench the fluorescence even further, higher antibody-conjugated GO concentrations could be less sensitive for sensing analyte tau at low concentrations. So there is a delicate balance between the GO concentration and the change in fluorescence signal. In the following experiment, antibody-conjugated GO always had a concentration of 100 µg ml^−1^.

### 3.3. Sensing of tau and the limit of detection

Based on these results mentioned above, we carried out quantitative sensing of analyte tau protein: a group of human tau protein samples ranging from 0 to 600 ng ml^−1^ were prepared in buffer solution and tested. In our experiment, the ‘blank’ sample consisted of antibody-conjugated GO and tau-FITC without analyte tau. To show the difference in fluorescence quenching upon addition of analyte, we always compared the fluorescence intensity of the blank without analyte versus sample with analyte.

**Figure 4.**
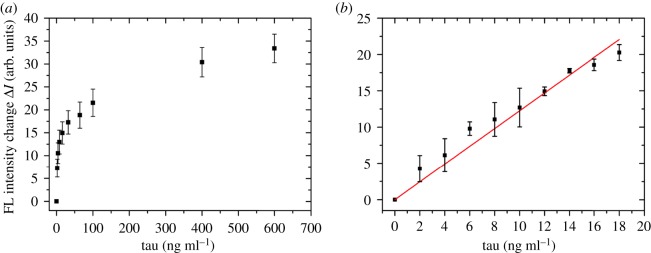
(*a*) The fluorescence (FL) intensity change (Δ*I*) of 100 ng ml^−1^ tau-FITC as a function of the increase in human tau at 0, 2, 4, 8, 16, 32, 64, 100, 400 and 600 ng ml^−1^; each sample contains 100 µg ml^−1^ rabbit anti-human tau antibody-conjugated GO. (*b*) The linear relationship between FL intensity (Δ*I*) and low tau concentrations (0, 2, 4, 6, 8, 10, 12, 14, 16, 18 ng ml^−1^) for the detection of LOD. The linear fitting has the following equation: Δ*I* = 1.23*c* (tau concentration); *R*² is 0.98888.

As mentioned above, the fluorescence-quenching efficiency is determined by energy coupling between the GO surface and the adsorbed tau-FITC. Surface modifications and the interfacial environment largely affect quenching efficiency. With 100 µg ml^−1^ of conjugated GO and 100 ng ml^−1^ of tau-FITC, the fluorescence intensity is approximately 200 at 520 nm. The addition of analyte tau will increase the signal starting from this relatively large background signal. To make the results directly proportional to analyte concentration, we use the change in intensity (Δ*I*) instead of *I*. Figure 4*a* shows an assay response curve prepared by this approach. The value of Δ*I* was calculated by subtracting the final fluorescence intensity at 520 nm and the ‘blank’ sample without analyte tau.

Figure 4*a* shows that the fluorescence signal increased readily with increasing analyte tau concentrations. The Δ*I* rapidly increased with analyte tau up to approximately 32 ng ml^−1^ after which the change began to plateau. The total change in signal reached a maximum at an analyte concentration of 600 ng ml^−1^.

The experiment was repeated five times, and the average results are plotted in figure 4*a*. Two main features could be observed from the plot: first, the initial rise in fluorescence signal is very rapid with a nearly linear region at concentrations below 20 ng ml^−1^; second, the signal change reached about half of the maximum value with only 32 ng ml^−1^ analyte concentration (versus that of 600 ng ml^−1^ at maximum).

The results indicate that the platform is feasible, and the analyte tau proteins could control the fluorescence quenching and thus regulate fluorescence intensity. We can generate a linear plot relating the tau-FITC fluorescence intensity with analyte tau concentrations from 0 to 20 ng ml^−1^ (figure 4*b*). Experiments under similar conditions were performed three times, and the data points in figure 4*b* were linear. The fitting function of the curve is Δ*I* = 1.23*c* (tau concentration) with an *R*² of 0.98888.

We then repeated the blank experiment 10 times to calculate the standard deviation (s.d.) of 10 background signals (electronic supplementary material, table S1). In our case, the blank means the experiment was performed the same as the sensing method mentioned above but without analyte tau; only tau-FITC and antibody-conjugated GO were involved to evaluate the intensity of the background. We took three times the value of the s.d. and divided it by the slope of 1.23 from the calibration curve to get a LOD of 6.4 ng ml^−1^ (0.14 pmol ml^−1^). While we assumed that tau-FITC proteins are all uniformly labelled with fluorescent FITCs, this may vary based on the quality of the antibodies and percentage of FITC labelling on tau [41]. Thus, the LOD could be improved theoretically with more controlled and uniform FITC labelling of tau. However, this is beyond the scope of this paper.

We compared our biosensor to other techniques (table 1). Our LOD is better than the electronic sensor (the LOD is 0.2 µmol ml^−1^ [42]) and the surface plasmon resonance (SPR) method (the LOD is 0.618 µmol ml^−1^ [45]). The LOD of the surface-enhanced Raman scattering (SERS) method is better than our work (0.025 fmol ml^−1^ [43] and 1.97 fmol ml^−1^ [44]). This is because the signal of the tau protein was amplified by the SERS effect. Compared with SERS, the LOD of our method was limited by the intensity of fluorescence emitted by FITC groups on tau protein. Since the molecular weight of tau protein is over 50 kDa [7], a concentration of tau-FITC ranging 0–100 pg ml^−1^ (the baseline level of tau concentration in human CSF [9,10]) means there are about 2 fmol ml^−1^ FITC groups in the solution, which is too low to be detected by fluorescence spectrophotometer. This is the main aspect that limits the current LOD of our method, which is approximately equal to the highest reported concentration of diseased state in human CSF [31]. An amplifying system of fluorescence is needed in this case in order to get a LOD ranging 0–100 pg ml^−1^, which will be explored in our future studies.

**Table 1.** A comparison of analysis method for the detection of tau protein.

technique	method	sample	detection	reference
electrochemistry	three electrode system	tau in PBS solution	0.2 µmol ml^−1^	[42]
SERS	sandwich assay of nanoparticles and Raman report	tau in PBS solution	0.025 fmol ml^−1^	[43]
SERS	nanoarchitecture-based 3D SERS platform	tau in PBS solution	0.15 ng ml^−1^ (1.97 fmol ml^−1^)	[44]
SPR	biosensor-based system	human serum	47 mg ml^−1^ (0.618 µmol ml^−1^)	[45]
fluorescence	GO-based competitive immunoassay	tau in PBS solution	6.4 ng ml^−1^ (0.14 pmol ml^−1^)	our method

### 3.4. Assay selectivity

**Figure 5.**
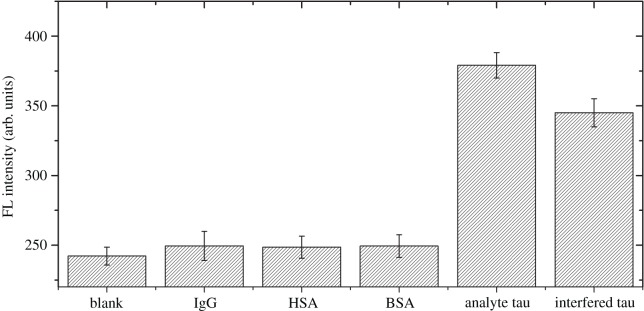
Fluorescence intensity of the assay in the presence of possible interfering molecules. The concentrations of IgG, HSA, BSA and analyte tau were all 200 ng ml^−1^. The tau-FITC was 100 ng ml^−1^, and the antibody-conjugated GO was 100 µg ml^−1^ for each experiment. The blank sample contained neither analyte tau nor interfering molecule. The sample named ‘interfered tau' contained 200 ng ml^−1^ of IgG, HSA and BSA as well as 10 ng ml^−1^ of analyte tau.

One major assumption is that tau proteins bind to antibody-conjugated GO via an antibody–antigen type of specific binding. This discriminative binding process means that the analyte tau will be adsorbed by antibody-conjugated GO preventing fluorescein labelled tau-FITC from binding to and quenching the surface. To confirm specificity, we performed selectivity assays. We added interfering molecules such as immunoglobulin G (IgG), human serum albumin (HSA) and BSA. These were introduced to test the selectivity. In this parallel testing, the signals from solutions containing analyte tau protein and blank sample without analyte tau were compared. The results (figure 5) clearly show that the three interfering biomolecules gave no detectable signal relative to blank. Only analyte tau protein showed a significant positive signal; the fluorescence intensity of other proteins was close to the blank. Analyte tau still had a positive signal even in the sample with analyte tau as well as IgG, HSA and BSA (concentration of the interfering protein is 20-fold higher than tau). This indicates that our assay design is highly selective for tau protein.

## 4. Conclusion

We developed an immunosensor based on fluorescence quenching between adsorbed fluorescent tau-FITC proteins and antibody-conjugated GO nanosheets. This signal was quantitatively controlled by the concentration of tau analyte. Competitive binding of analyte tau and standard tau-FITC on the GO surface's limited binding sites modulates free tau-FITC in solution and hence signal. The increase in fluorescence signal (from tau-FITC) correlates directly with increasing tau concentration. The assay based on antibody-conjugated GO does not require any phase separation steps or wash steps to remove unadsorbed antibodies as in commercial ELISA procedures. Thus, our assay benefits from fewer reagents, lower cost, and less systematic and human errors. The LOD is about 0.14 pmol ml^−1^, which could be improved further. The sensing mechanism in this work could become a viable immunosensor platform for the detection of tau protein and may be useful in the clinical diagnosis of AD or other tau-induced neurodegeneration syndromes.

## Supplementary Material

Controlled Fluorescence Quenching by Antibody-conjugated Graphene Oxide to Measure Tau Protein

## Data accessibility

The datasets supporting this article have been uploaded as part of the electronic supplementary material.
