# Supplementary material for: Controlled fluorescence quenching by antibody-conjugated graphene oxide to measure tau protein
Source: R Soc Open Sci. 2018 Apr 11;5(4):171808. doi: 10.1098/rsos.171808 (PMC5936912; doi:10.1098/rsos.171808)
Supplement: Controlled Fluorescence Quenching by Antibody-conjugated Graphene Oxide to Measure Tau Protein [file rsos171808supp1.docx]

Supporting Materials

**C****ontrolled Fluorescence Quenching by Antibody-conjugated Graphene Oxide to Measure Tau Protein**

**Ao Huang, Luning Zhang, Weiwei Li, Zeyu Ma, Shuo Shi* and Tianming Yao***

School of Chemical Science and Engineering, Tongji University, 1239 Siping Rd, Shanghai, 200092,PR China.

**The infrared spectrum of graphene oxide**

In order to check the existence of carboxyl, graphene oxide (GO) was characterized by infrared spectrum using a Fourier transform infrared spectroscopy (Thermo Scientific Nicolet IS10). Briefly, 1 mg GO was dispersed in 2 g potassium bromide. The mixture was pulverized and pressed like a pellet. Then, the pellet was set into the fourier transform infrared spectroscopy and detected. The result was shown in Figure S1.


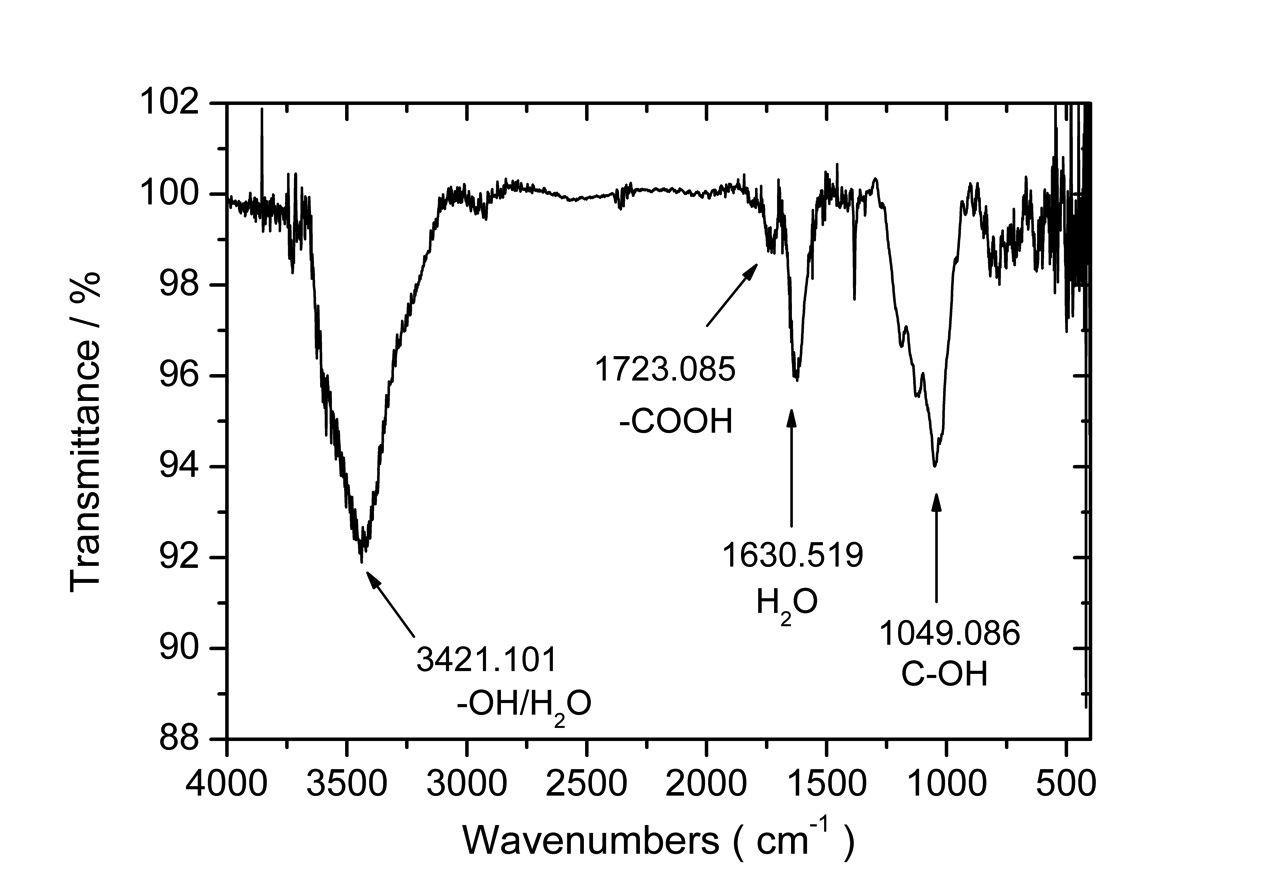


**Figure S1** The infrared spectrum of GO

4 main peaks were assigned to the GO sample: hydroxyl of water (3421 cm^-1^), carboxyl (1723 cm^-1^), water (1630 cm^-1^) and alcoholic hydroxyl (1049 cm^-1^). The results shown that our GO sample did have carboxyls.

**The surface modification of GO by antibodies**

In traditional ELISA methods, the antibodies were physically adsorbed by polystyrene plates. In order to make the antibodies adsorbed as many as possible, the amount of antibodies were usually excessive. We followed this commercial custom and used over amount antibodies in synthesizing antibody-conjugated GO. To measure the exactly quantity of antibodies on GO surface, we use an increasing concentration of fluorescein conjugated antibodies to react with a series of EDC-NHS activated GO samples with constant concentration. Briefly, an increasing amount (0, 2, 4, 8, 16, 32, 64, 100, 200, 400 μg mL^-1^) of FITC conjugated antibodies were added in 10 centrifuge tubes containing 100 mg EDC-NHS activated GO each. After 1h, 2% BSA solution was added to each sample to block the remaining binding sites, then the fluorescence intensity of which was detected. If FITC labelled antibodies were conjugated to GO surface, the fluorescence would be quenched. Compare the fluorescence intensity of these FITC labelled antibody conjugated GO with other samples containing only FITC labelled antibodies in the same concentration; the immobilization amount of antibodies on GO surface could be measured (Figure S2).


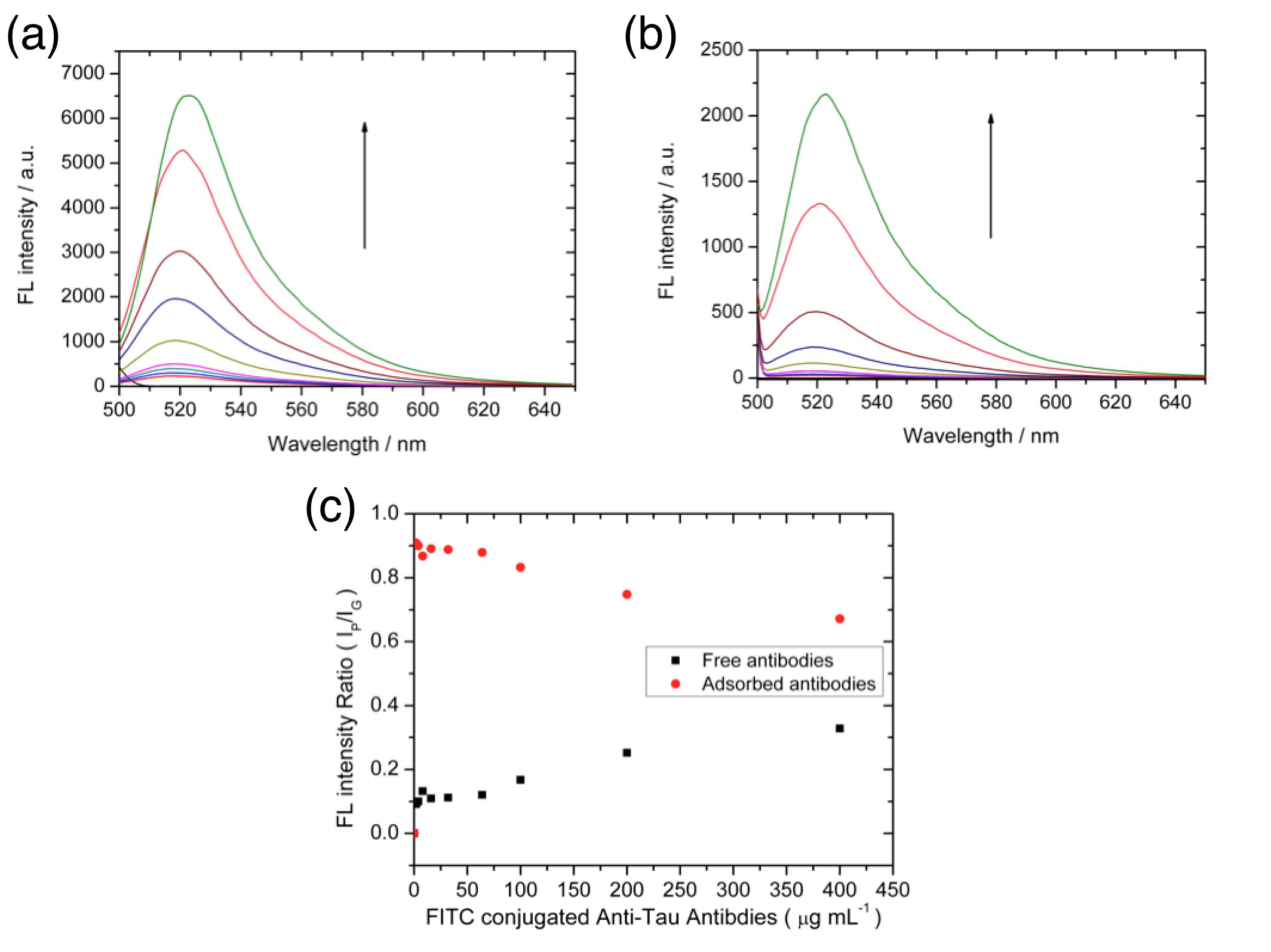


**Figure S2** The immobilization of antibodies on GO surface. (a) The fluorescence intensity (I_P_) of FITC labelled antibodies of 0, 2, 4, 8, 16, 32, 64, 100, 200, 400 μg mL^-1^. (b) The fluorescence intensity (I_G_) of 100 μg mL^-1^ GO coupling 0, 2, 4, 8, 16, 32, 64, 100, 200, 400 μg mL^-1^ FITC labelled antibodies. (c) The ratio of I_P_ / I_G_ with the increasing of FITC labelled antibodies. This ratio indicates the ratio of adsorbed antibodies. The "free antibodies" mentioned by the graph were predicted by the equation 1- I_P_ / I_G._

Comparing Figure S2 (a) and Figure S2 (b), we could find the fluorescence intensity of Figure S2 (b) is much lower in order to the presence of GO. Figure S2 (c) shown when the concentration of antibodies is beyond 0~64 μg mL^-1^, almost 90% antibodies were adsorbed on GO surface. In our work, 50 μg mL^-1^ antibody was used in EDC-NHS coupling reaction. With the result showned in Figure S2 (c) we could predicted that most of these antibodies were conjugated on GO surface.

**About the little peak of 605 nm on fluorescence spectrum**

In order to proof the peak of 605 nm was a result of scattering, we made a sample following all the steps of our route, and detect the fluorescence intensity both before and after centrifuge. In our original work, the sample was not centrifuged before detecting.


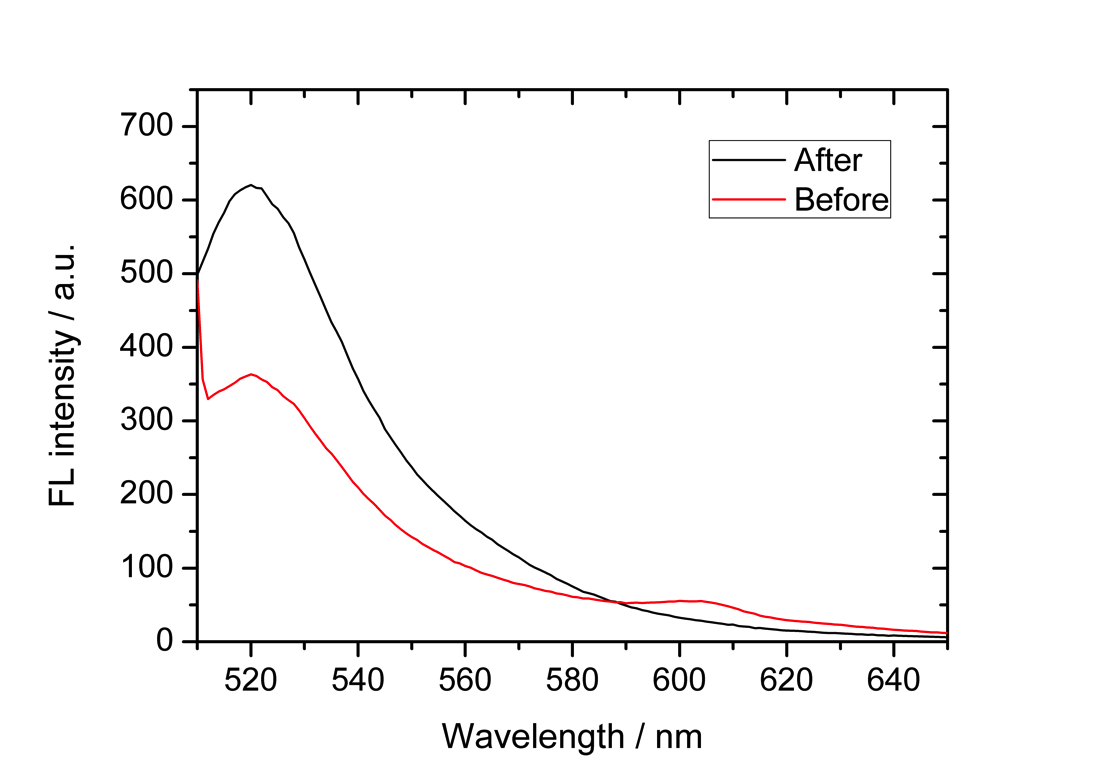


**Figure S3** The fluorescence spectrum before and after centrifuge. The concentration of analyte Tau, Tau-FITC and antibody-conjugated GO were 10 ng mL^-1^, 100 ng mL^-1^ and 100 μg mL^-1^

As Figure S3 indicated, there was a peak at 605 nm before the sample was centrifuged. After the sample was centrifuged, the peak disappeared. The fluorescence intensity also became higher after the sample was centrifuged, for GO sheets were no longer present in the upper solution.The upper soluton became transparent and emitted higher fluorescence intensity without the block of GO. From this result we could proof the peak 605 nm is scattering.

**The temperature of immune reaction**

Theoritically, immune reaction could be performed at room temperature. We perform the immune reaction at 37 ℃ on the purpose of simulating the human inner environment, the temperature affects little on immune reaction. We prepared two sample with the same concentration of analyte Tau, Tau-FITC and antibody-conjugated GO. One was keeped at 37 ℃ while performing immune reaction, the other was keeped at room temperature(Figure S4). Both sample were detected at the room temperature.


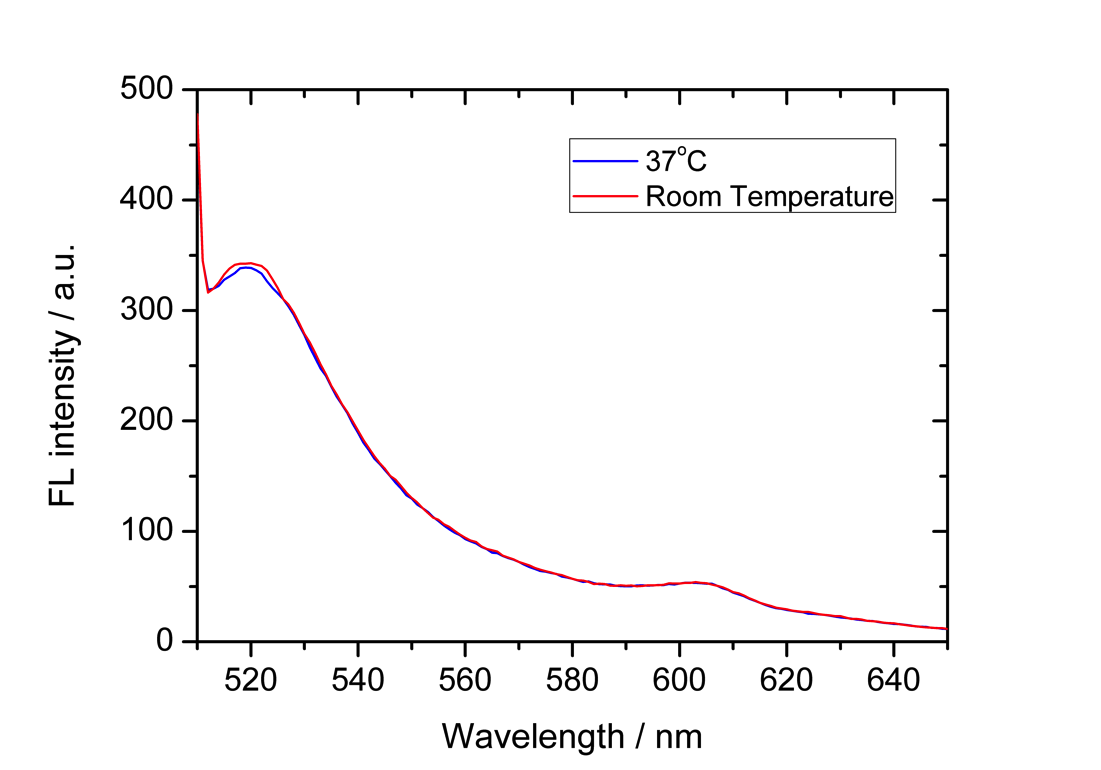


**Figure S4** The fluorescence intensity of two sample. One was keeped at 37 °C，the other was keeped at room temperature. The cencentration of analyte Tau, Tau-FITC and antibody-conjugated GO in both sample were 10 ng mL^-1^, 100 ng mL^-1^ and 100 μg mL^-1^

From the result shown in Figure S4 we found the fluorescence intensity of both sample was nearly the same.

**About the limit of detection**

We measured 10 blank samples and calculated the standard deviation shown in table 1 of our text. We took threefold I/I_0_ value of the SD and divided it by the slope of the calibration curve and got the LOD of 0.14 pmol mL^-1^.

| 10 blank samples | | | | | | | | | | SD |
| --- | --- | --- | --- | --- | --- | --- | --- | --- | --- | --- |
| 215.3 | 215.6 | 216.1 | 216.1 | 218.4 | 219.9 | 220.6 | 220.9 | 221.8 | 222.5 | 2.621373686 |

**Table S1** SD of 10 blank samples.

LOD=SD*3/slope/mole weight= 2.621373686*3/1.23/46000=0.14 pmol mL^-1^. In this function, 2.621373686 is the SD, 1.23 is the slope of the calibration curve ( ΔI = 1.23c(tau concentration)) and 46000 is the mole weight of Tau.
